# Can the Norton Scale Score Be Used as an Adjunct Tool for Implantable Defibrillator Patient Selection? A Retrospective Single-Center Cohort Study

**DOI:** 10.3390/jcm12010214

**Published:** 2022-12-27

**Authors:** Shir Ben Asher Kestin, Ariel Israel, Eran Leshem, Anat Milman, Avi Sabbag, Ilan Goldengerg, Eyal Nof, Roy Beinart

**Affiliations:** 1 Sackler Faculty of Medicine, Tel-Aviv University, Tel-Aviv 6997801, Israel; 2 Department of Internal Medicine C, Meir Medical Center, Kfar-Saba 4428164, Israel; 3 Leumit Research Institute, Leumit Health Services, Tel-Aviv 647378, Israel; 4 Leviev Heart Center, Sheba Medical Center, Ramat Gan 5266202, Israel; 5 Department of Medicine, School of Medicine and Dentistry, University of Rochester Medical Center, Rochester, NY 14642, USA

**Keywords:** sudden cardiac death, implantable cardioverter device, Norton scale, pressure ulcer, patient selection

## Abstract

(1) Background: Implantable cardioverter defibrillators (ICDs) have become the standard of care in the prevention of sudden cardiac death, yet studies have shown that competing causes of death may limit ICD benefits. The Norton scale is a pressure ulcer risk score shown to have prognostic value in other fields. The purpose of this study was to assess the use of the Norton scale as an aid for ICD patient selection; (2) Methods: The study was comprised of consecutive patients who underwent defibrillator implantation at Sheba Medical Center between 2008 and 2016. A competing risk analysis was performed to assess the likelihood of death prior to device therapy; (3) Results: 695 patients were included. A total of 59 (8.5%) patients had low admission Norton scale score (ANSS) (≤14), 81 (11.7%) had intermediate ANSS (15–17), and the remainder (79.8%) had high (18–20) ANSS. The cumulative probability of all-cause mortality within one year of ICD implantation in patients with low ANSS was 30%, compared with 20% and 7% among the intermediate- and high-ANSS groups, respectively. Moreover, the one-year mortality rate without ICD therapy in low-ANSS patients was over four-fold compared with that of high-ANSS patients (33% versus 7%, *p* < 0.0001); (4) Conclusions: The Norton scale could be a useful additional tool in predicting the life expectancy of ICD candidates, thereby improving patient selection.

## 1. Introduction

Implantable cardioverter defibrillators (ICDs) have been previously shown to prevent sudden cardiac death in patients with reduced LV function [1,2] and in patients with a history of life-threatening arrhythmia or aborted sudden cardiac death [3]. ICD therapy is currently recommended in various indications by the ESC and AHA/ACC/HRS [4,5,6]. These guidelines, however, mandate that candidates for ICD implantation have a life expectancy of at least one year, with a reasonable functional status. In practice, it is difficult for clinicians to predict the survival of patients with heart failure [7,8,9,10]. Consequentially, current patient selection for ICD implantation is challenging and remains sub-optimal, despite decades of experience. In fact, most patients who receive an ICD for primary prevention die of non-cardiac causes without utilizing life-saving shock therapy [10,11]. These findings suggest a need for improved methods for the prediction of life expectancy and better patient selection for ICD implantation.

Improper patient selection for ICD implantation imposes a considerable burden on health systems worldwide and exposes patients to unnecessary procedures and related complications [7,12].

Various stratification scales have been suggested as means to identify suitable candidates for defibrillator implantation [12,13,14]; however, none have been widely accepted in practice. Frailty has been shown to correlate with poor outcomes after ICD implantation [15], but the definition of frailty is non-uniform and it is assessed without consensus.

The Norton scale, originally purposed and used as a tool for pressure ulcer risk assessment, is routinely used by nursing staff in inpatient settings across the world, [16,17]. The scale evaluates five functional and clinical criteria (physical condition, mental condition, activity, mobility, and incontinence). For each criterion, the patient is given a score ranging from one to four, adding up to a total of five to twenty, with a lower score representing a higher risk (Table S1). The use of basic functional parameters contributes not only to its ease of use, but also to the applicability of the Norton scale to other fields, as it makes it indicative of the patients’ basic general condition. Together with the fact that it is routinely calculated for patients on admission, the Norton scale appears to be an attractive tool for other stratification targets. Interestingly, the Norton scale has been suggested as a useful prognostic tool in many other disciplines, including cardiology [18,19]. The aim of this study was to evaluate the usefulness of the admission Norton scale score (ANSS) in identifying patients in whom ICD implantation would be futile, as their life expectancy would be less than one year.

## 2. Materials and Methods

Our study comprised a cohort of consecutive adult patients who underwent ICD or Cardiac Resynchronization Therapy with a Defibrillator (CRT-D) implantation or generator replacement at the Chaim Sheba Medical Center between January 2008 and June 2016. The routine device programming is detailed in Table S2. Following the approval of the institutional review and ethics board, patient data were retrospectively extracted from the electronic medical records. Patient records were maintained using a Chameleon^®^ information system (Elad Healthcare Solutions).

The Norton score was computed at the time of admission by registered nurses using a dedicated computerized form as part of the mandatory admission protocol for all inpatients. Data collection was performed using Structured Query Language and custom Python/Pandas scripts accessing the Chameleon database. All patients aged 18 or above who underwent an ICD/CRT-D implantation or generator replacement during the study period were included.

The main study outcomes were all-cause mortality, appropriate defibrillation therapy, and death prior to defibrillation therapy. Data regarding defibrillator therapy were extracted from the devices during the regularly planned follow-up visits. Death events were queried from the Israel national population registry, with each patient identified by their unique national ID number. There were no patients lost to follow-up. The median follow-up time was 35 (IQR 22–49) months.

For descriptive statistics, we used the T-test to evaluate the statistical significance of differences in ordinal features distributed normally, the Kruskal–Wallis Rank Sum Test for non-normally distributed variables, and Fisher’s exact test to compare categorical features. We used Kaplan–Meier’s method to assess survival according to three pre-defined ANSS groups: low (≤14), intermediate (15–17), and high (18–20). Subgroup analyses were performed for patients with a reduced left ventricular ejection fraction (LVEF) (≤35%) and for patients with high vs. low-risk clinical features (age ≥ 70 and/or creatinine ≥ 1.5 mg/dL) [20,21]. We further used COX proportional hazard models to calculate predictors of mortality in univariable models and multivariable models adjusted for age, gender, and comorbidities (CHF, Renal Disease, diabetes mellitus, and prior CVA); for multivariable models, we used backward stepwise selection to keep only significant predictors in the displayed models. Death without prior ICD therapy was assessed using a competing risk analysis among patients that did not receive shock therapy, i.e., patients who received shock were censored at the date of their first shock therapy and not further included in the population at risk. All statistical analyses were performed in R Statistical Language (R 3.5.1). *p* < 0.05 was considered significant.

## 3. Results

### 3.1. Cohort Population

Among the 695 study patients, the mean age was 66 ± 14 years, 85% were males, and the mean LVEF was 32 ± 14%. Sixty-six percent underwent de-novo defibrillator implantations.

**Figure 1:**
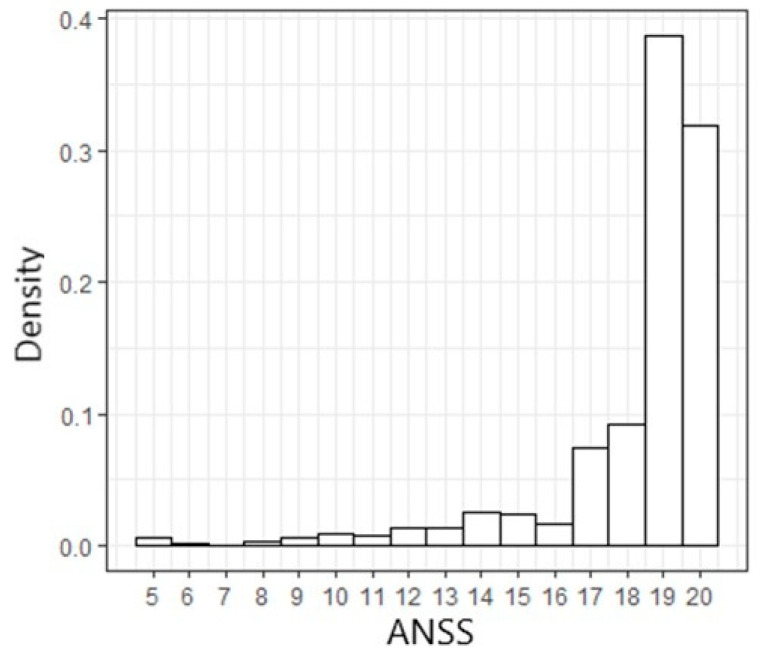
Distribution of admission Norton scale scores in the study population.

The cohort was divided into three pre-specified ANSS groups as routinely used in practice [22]: low (≤14), intermediate (15–17), and high (18–20) (Table S1). Due to the low number of patients with very low ANSS (<10), this group was included in the low (≤14) group. Most of the study population comprised the high-score group (79.8%), creating a leftward skew in the ANSS distribution, with a median of 19 (IQR 18–20) (Figure 1). A total of 59 patients (8.5%) had a low ANSS and 81 patients (11.7%) had an intermediate ANSS.

The baseline characteristics of the study population are shown in Table 1. Patients with lower ANSS were older and had significantly higher rates of heart failure, diabetes mellitus, and renal dysfunction, and a history of cerebrovascular accidents. Moreover, they had higher creatinine levels, as well as lower albumin and hemoglobin levels, representing their poor general condition. No major differences in medical treatment were noted.

### 3.2. All-Cause Mortality

Throughout the study period, 174 patients died; of those, 75 (43%) died within the first year following device implantation. In fact, the one-year mortality was significantly higher in patients with low ANSS (18 (30.5%)) compared with those with intermediate ANSS (16 (19.8%)) and with high ANSS (41 (7.4%)), (*p* < 0.001). The results were also consistent at two years after implantation: 27 (45.8%) patients from the low-ANSS group versus 22 (27.2%) and 74 (13.3%) patients from the intermediate- and high-ANSS groups, respectively (*p* < 0.001).

**Figure 2:**
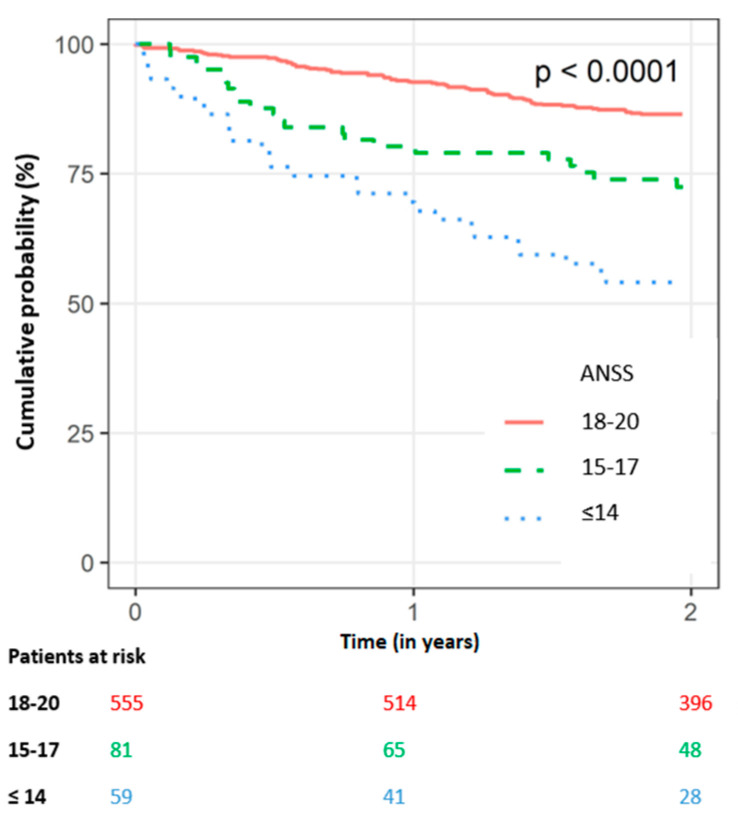
Kaplan–Meier Survival curve for all-cause mortality over time in the study population by admission Norton scale score. (Numbers reflect patients at risk. *p* value < 0.0001.) (ANSS, Admission Norton scale score).

The Kaplan–Meier survival analysis demonstrates a statistically significant difference in the all-cause mortality at one year amongst the three Norton score groups, with the highest cumulative probability in the low-ANSS group (30%), compared with 20% and 7% among those in the intermediate- and high-ANSS groups, respectively. Likewise, at the two-year follow-up, the respective mortality probability rates were 46%, 27%, and 13% (log-rank *p*-value < 0.001 for the overall difference during follow-up; Figure 2).

### 3.3. Patients Not Receiving Appropriate ICD Therapy

**Figure 3:**
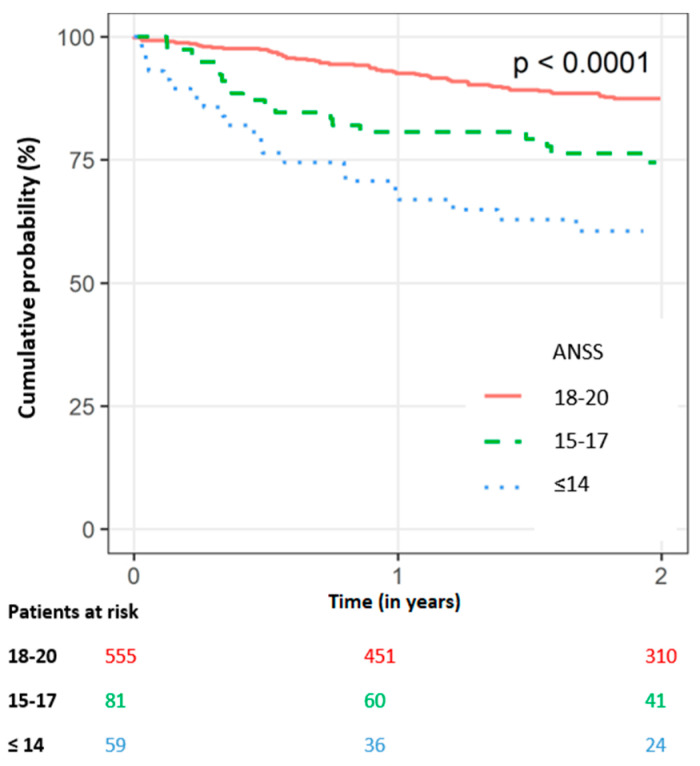
Kaplan–Meier survival curve for death without prior defibrillator therapy over time by admission Norton scale score (numbers reflect patients at risk. *p* value < 0.0001). (ANSS, Admission Norton scale score).

We further assessed the risk of death for patients who did not receive appropriate ICD therapy at any time following implantation. At one year following implantation, 70 patients died without ICD therapy: 17 (28.8%) patients from the low-ANSS group versus 15 (18.5%) and 38 (6.8%) patients from the intermediate and high groups, respectively (*p* < 0.001). Similarly, at two years, 101 patients died without ICD therapy: 21 (35.6%) patients versus 19 (23.5%) and 61 (11%) patients, respectively (*p* < 0.001). Kaplan–Meier analysis showed a statistically significant association between mortality without ICD therapy and decreasing Norton score (Figure 3). The one-year probability of death without any ICD therapy following implantation among patients in the low-ANSS group was over four-fold higher compared with those with high ANSS (33% versus 7%). Similar findings were evident at two years following implantation (40% versus 12%) (log-rank *p*-value < 0.0001 for the overall difference during follow-up).

### 3.4. Association with Known Predictors of Poor Prognosis

Additionally, we evaluated the usefulness of ANSS in association with gender, age, and factors known to be associated with poor prognosis (congestive heart failure, diabetes mellitus, renal disease, and prior cerebrovascular accident) and performed a multivariate survival analysis using the Cox proportional hazards model adjusting for these variables. Our analysis demonstrates that, after adjusting to these significant factors, ANSS maintained its independent value. Patients in the low-ANSS group had a substantially higher risk of death from any cause compared with those in the high-ANSS group (HR: 2.39; 95% CI: 1.6–3.6; *p* < 0.001). Similarly, patients with intermediate ANSS had a higher risk of all-cause mortality compared with patients with high ANSS. (HR: 1.57; 95% CI: 1.03–2.39; *p* = 0.036) (Table 2). Likewise, the adjusted risk of death without prior ICD therapy was significantly higher in the low-ANSS group, and higher, albeit borderline significant, in the intermediate-ANSS group compared with the high-ANSS group (HR: 2.29; *p* = 0.001, and HR: 1.58; *p* = 0.06, respectively) (Table 3).

### 3.5. Subgroup Analysis

Additional analysis was performed for a subgroup of 448 patients with known LVEF ≤ 35%, representing the group of patients receiving ICD as indicated for cardiomyopathies and poor left ventricular function (Table S3). A Kaplan–Meyer analysis (Figures S1 and S2) yielded consistent results and showed higher mortality rates among patients with low ANSS (cumulative probabilities of all-cause mortality within one year for low versus high ANSS were 30% and 9% respectively, log rank *p* < 0.0001; for death without ICD therapy, the probabilities were 30% vs. 8%, respectively; log rank *p* < 0.0001).

**Figure 4:**
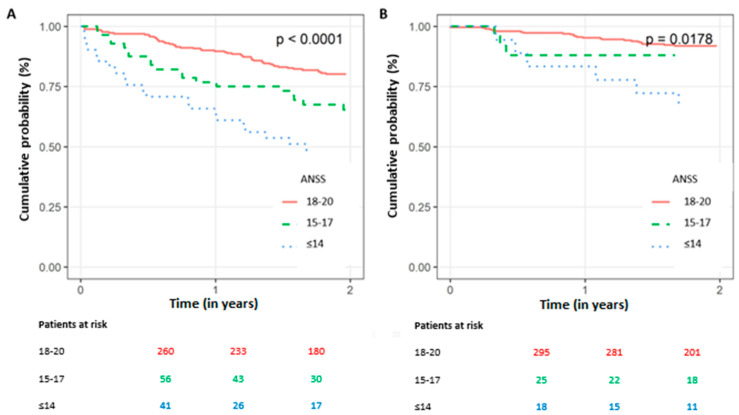
Kaplan–Meier survival curve for all-cause mortality over time in high vs. low clinical risk patients by admission Norton scale score. (**A**) High-clinical-risk patients: ≥70 years old and/or baseline serum creatinine values ≥ 1.5 mg/dL. *p* value < 0.0001. (**B**) Low-clinical-risk patients: <70 years old and baseline serum creatinine values < 1.5 mg/dL. *p* value = 0.0178. Numbers reflect patients at risk. ANSS, admission Norton scale score.

We explored the utility of ANSS for risk stratification in patients with pre-defined high or low risk of mortality based on known clinical factors [20,21]. This sub-analysis showed that low ANSS remained a significant marker of increased all-cause mortality among patients with a high clinical risk of death (i.e., ≥70 years old and/or with baseline serum creatinine values ≥ 1.5 mg/dL), as well as in those with low clinical risk (i.e., <70 years old and with baseline serum creatinine values < 1.5 mg/dL) (Figure 4A,B). Notably, patients who had both a low ANSS and a high clinical risk experienced mortality rates at one year approaching 40% (Figure 4A). When applied to the subgroup of patients with LVEF ≤ 35%, our results remained consistent (Figure S3A,B).

## 4. Discussion

Our findings show that patients with low ANSS were significantly more likely to die of any cause within one year of ICD implantation compared with high-ANSS patients. Moreover, they had a higher probability of dying without having received appropriate device therapy.

ICDs have become a fundamental part of the current guidelines for the prevention of sudden cardiac death in the setting of both primary and secondary prevention [4,5,23]. Nonetheless, the current guidelines require a life expectancy substantially over one year with reasonable functional status, a challenging decision for clinicians in the real-life setting [7,8]. Recent studies have shown that a high percentage of ICD recipients do not benefit from the device in real-life settings and, in fact, die of non-cardiac causes [10,11,24]. Improper patient selection imposes a considerable burden on health systems worldwide and exposes patients to unnecessary procedures and complications, such as infections and inappropriate device therapy [7,12,25], emphasizing the need for tools assisting in the identification of those who will not benefit from ICD therapy.

Previous studies have suggested various factors as possible markers for reduced survival following ICD implantation [12,20,21]. However, no single marker has been found to be as robust as LVEF [9]. In a recent observational study by Garcia et al. [26], regarding ICD for primary prevention, the combination of older age (≥70), NYHA class ≥ III, and AF was associated with a 22.63% cumulative risk of mortality within one year of ICD implantation, but not as single markers. Similarly, different scoring systems and algorithms for the prediction of mortality in ICD-eligible patients have been suggested [13,14,27,28,29,30], but most are complex and none are currently used in practice. Our study suggests that the Norton scale, a simple and widely used pressure ulcer risk-assessment scale [16,17], could have high discriminative value for the prediction of non-cardiac mortality following ICD implantation. This scale has been previously shown to predict outcomes following trans-aortic valve implantation and myocardial infractions [18,19]. To our knowledge, it has not been used as a predictor of mortality in ICD recipients.

In this study, the cohort was divided into three prespecified ANSS groups (high risk, ANSS ≤ 14; medium risk, ANSS 15–17; and low risk, ANSS ≥ 18). We found that patients with ANSS ≤ 14 were significantly more likely to die of any cause for one year following ICD implantation and over four-fold more likely to die without having received appropriate device therapy compared with patients with ANSS ≥ 18. Our results remained significant at two years of follow-up and were validated by logistic regression models, proving the strength of the ANSS as a prognostic tool for ICD-implanted patients.

The endpoint of this study was all-cause mortality, and the cause of death was not specified, as mortality of any cause is the limiting factor for ICD implantation. Nevertheless, it would be of interest for further studies to focus on the relationship between the cause of death of these patients and ANSS. Of note, 75 patients (10.8% of the cohort) died within one year of device implantation, as compared with 6% for SCD-Heft [2] and 8% for MADIT-II [1]. Two recent studies regarding primary-prevention ICD had one-year mortality rates of 4.2% [26] and 4.8% [29]. Accurate rates of one-year mortality following ICD implantation are lacking, and great variation exists depending on the indication for implantation and etiology. Our population is greatly heterogeneous, as it is based on a single-center database and includes all implantations and replacements in the follow-up period. The fact that this study did not discriminate between de-novo implantations and device replacements is a limitation that requires further studies to address the value of the ANSS in these different patient populations. Still, a one-year life expectancy is required for all ICD recipients, and the requirements should ideally be re-evaluated before any device replacement, making this study relevant for the decision-making process.

Another limitation is that different ventricular fibrillation zone cut-offs were used according to the physicians’ preferences and nominal values as determined by various vendors. This could possibly affect the rates of ICD therapies. Nevertheless, our primary finding of higher one-year mortality rates in ICD recipients with low ANSS should not be affected significantly by such nominal programming.

A recent systematic review by Chen et al., found that frailty in older patients, as assessed by various tools, is associated with a higher risk of mortality after ICD implantation for primary prevention [15], and various studies have shown that older age and renal failure are associated with poor outcomes following ICD implantation [20,21,31,32]. We, therefore, further explored the discriminative utility of the ANSS in these high-risk populations. Indeed, ANSS maintained its prognostic value in elderly patients and/or in patients with renal dysfunction. Hence, ANSS has independent prognostic value for survival, even in ICD recipients with high clinical risk, making it a potential supporting tool in the prediction of life expectancy and functional status for ICD candidates.

Our results demonstrate the possible value of ANSS in the assessment of ICD candidates having the potential to help and avoid futile implantation of defibrillators in patients with an ANSS of 14 or less. It showed that the one-year probability of death without ICD therapy in the low-ANSS group was over four-fold higher compared with that of the high-ANSS group, yet approximately two-thirds of patients did not die at one year without receiving ICD therapy. We suggest that combining the ANSS into the SCD-prevention decision-making process could provide support for clinicians in a real-life setting, but not as a stand-alone tool. Further prospective and randomized controlled studies are needed to validate these initial findings. If proven to have strong prognostic value in the appropriate populations in future studies, the use of the Norton scale could even be considered for incorporation into the guidelines for SCD prevention and assist in standardizing the currently ambiguous requirement for a life expectancy of at least one year.

## 5. Conclusions

Current guidelines for ICD implantation recommend a life expectancy of one year with reasonable functional status. The results of this study suggest that ANSS could be useful in the prediction of life expectancy in ICD candidates and might be used as a supportive tool, improving patient selection for ICD implantation.

## Figures and Tables

**Table 1:** Baseline characteristics by admission Norton scale score.

Clinical Characteristics	Overall	ANSS ≤ 14	ANSS 15–17	ANSS 18–20	*p* Value
Study Population	*n* = 695	*n* = 59	*n* = 81	*n* = 555	
Age at procedure (years) ± SD	66 ± 14	70 ± 14	70 ± 12	65 ± 14	0.001
Female (%)	106 (15)	10 (17)	14 (17)	82 (15)	0.783
ANSS (median (IQR))	19 (18–20)	12 (10–14)	17 (16–17)	19 (19–20)	<0.001
High clinical risk * (%)	357 (51)	41 (70)	56 (69)	260 (47)	<0.001
De-novo ICD implantation (%)	461 (66)	42 (71)	52 (65)	367 (67)	0.734
Prior myocardial infarction (%)	262 (38)	19 (32)	33 (41)	210 (38)	0.546
Congestive heart failure (%)	345 (50)	37 (62)	49 (61)	259 (47)	0.008
Atrial fibrillation (%)	200 (29)	22 (37)	28 (35)	150 (27)	0.128
Prior CVA (%)	80 (12)	17 (29)	16 (20)	47 (9)	<0.001
Prior TIA (%)	19 (0.03)	1 (2)	1 (1)	17 (3)	0.560
Dyslipidemia (%)	321 (46)	28 (48)	37 (46)	256 (46)	0.990
Currently on dialysis (%)	3 (0.004)	1 (2)	1 (1)	1 (0.2)	0.123
Hypertension (%)	358 (52)	31 (53)	47 (59)	280 (51)	0.430
Diabetes mellitus (%)	235 (34)	29 (49)	35 (44)	171 (31)	0.003
Smoker (%)	165 (24)	13 (22)	19 (24)	133 (24)	0.931
BMI (kg/m^2^), ±SD	27 ± 5	27 ± 6	27 ± 5	27 ± 5	0.707
GFR MDRD (mL/min/1.73 m^2^), ±SD	65 ± 32	66 ± 59	61 ± 26	66 ± 28	0.462
Serum creatinine (mg/dL), ±SD	1.3 ± 0.7	1.5 ± 1	1.4 ± 0.9	1.3 ± 0.6	0.024
Hemoglobin (g/dL), ±SD	13 ± 2	11 ± 2	12 ± 2	13 ± 2	<0.001
Serum albumin (g/dL), ±SD	4 ± 0.5	3 ± 0.6	4 ± 0.5	4 ± 0.4	<0.001
LV ejection fraction (%), ±SD	32 ± 14	33 ± 14	32 ± 14	32 ± 14	0.851
ACE inhibitors (%)	453 (65)	39 (66)	51 (63)	363 (65)	0.900
Aldosterone antagonists (%)	422 (61)	37 (63)	58 (72)	327 (59)	0.087
Beta-blockers (%)	185 (27)	17 (29)	17 (21)	151 (27)	0.459
Antiarrhythmics:					
Class IB (%)	27 (4)	3 (5)	4 (5)	20 (4)	0.746
Class IC (%)	23 (3)	2 (3)	3 (4)	18 (3)	0.976
Class III (%)	330 (47)	35 (59)	36 (44)	259 (47)	0.152
Salicylic acid (%)	13 (2)	0 (0)	1 (1)	12 (2)	0.458

* High clinical risk defined as age ≥ 70 years and/or baseline serum creatinine values ≥ 1.5 mg/dL. Values are the mean ± standard deviation, median (IQR), or *n* (%). ACE, angiotensin-converting enzyme; ANSS, admission Norton scale score; BMI, body mass index; CVA, cerebrovascular accident; GFR, glomerular filtration rate; ICD, implantable cardioverter defibrillator; LVEF, left-ventricular ejection fraction; MDRD, modification of diet in renal disease; TIA, transient ischemic attack.

**Table 2:** COX proportional hazards univariable and multivariable models for all-cause mortality.

	Univariable Model			Multivariable Model		
	HR	95% CI	*p*	HR	95% CI	*p*
Age (year)	1.03	1.02–1.05	<0.001	1.02	1.00–1.03	0.018
Gender (Female)	0.73	0.46–1.16	0.179			
CHF	2.98	2.14–4.14	<0.001	2.15	1.52–3.04	<0.001
DM	1.66	1.23–2.25	<0.001			
Renal Disease	2.85	2.11–3.84	<0.001	1.89	1.37–2.61	<0.001
Prior CVA	1.54	1.02–2.32	0.040			
ANSS						
High (18–20) (reference)	1			1		—
Intermediate (15–17)	1.78	1.17–2.70	0.007	1.57	1.03–2.39	0.036
Low (≤14)	3.21	2.15–4.79	<0.001	2.39	1.59–3.60	<0.001

ANSS, admission Norton scale score; CHF, congestive heart failure; CVA, cerebrovascular accident; DM, diabetes mellitus.

**Table 3:** COX proportional hazards univariable and multivariable models for death without prior ICD therapy.

	Univariable Models			Multivariable Model		
	HR	95% CI	*p*	HR	95% CI	*p*
Age (year)	1.04	1.03–1.06	<0.001	1.02	1.01–1.04	0.010
Gender (Female)	0.68	0.40–1.17	0.163			
CHF	3.31	2.23–4.89	0.001	2.22	1.47–3.36	<0.001
DM	1.76	1.25–2.48	0.001			
Renal Disease	3.37	2.38–4.75	<0.001	2.12	1.46–3.07	<0.001
Prior CVA	1.69	1.08–2.65	0.023			
ANSS						
High (18–20) (reference)	1	—	—	1	—	—
Intermediate (15–17)	1.88	1.17–3.00	0.009	1.58	0.98–2.53	0.058
Low (≤14)	3.27	2.06–5.18	<0.001	2.29	1.44–3.77	0.001

ANSS, admission Norton scale score; CHF, congestive heart failure; CVA, cerebrovascular accident; DM, diabetes mellitus.

## Data Availability

Data are available upon request.

## Supplementary Materials

The following supporting information can be downloaded at: https://www.mdpi.com/article/10.3390/jcm12010214/s1, Figure S1: Kaplan–Meier Survival curve for all-cause mortality for patients with LVEF ≤ 35% over time by admission Norton scale score. Numbers reflect patients at risk. *p* value < 0.0001. ANSS = Admission Norton scale score; Figure S2: Kaplan–Meier Survival curve for death without prior defibrillator therapy in patients with LVEF ≤ 35% over time by admission Norton scale score. Numbers reflect patients at risk. *p* value < 0.0001. ANSS = Admission Norton scale score; Figure S3: Kaplan–Meier Survival curve for all-cause mortality over time in high vs. low clinical risk patients with LVEF ≤ 35%, by admission Norton scale score. Numbers reflect patients at risk. ANSS = Admission Norton scale score; Table S1: The Norton Pressure Sore Risk-Assessment Scale Scoring System; Table S2: Routine programming of implantable defibrillators in the Sheba medical center, Tel-Hashomer; Table S3: Baseline characteristics of patients with LVEF ≤ 35% by admission Norton scale score.
