# Supplementary material for: Can the Norton Scale Score Be Used as an Adjunct Tool for Implantable Defibrillator Patient Selection? A Retrospective Single-Center Cohort Study
Source: J Clin Med. 2022 Dec 27;12(1):214. doi: 10.3390/jcm12010214 (PMC9821316; doi:10.3390/jcm12010214)
Supplement: Supplementary file 1 [file jcm-12-00214-s001.zip › jcm-2052927-supplementary.pdf]

Supplementary Materials

Supplementary Figures

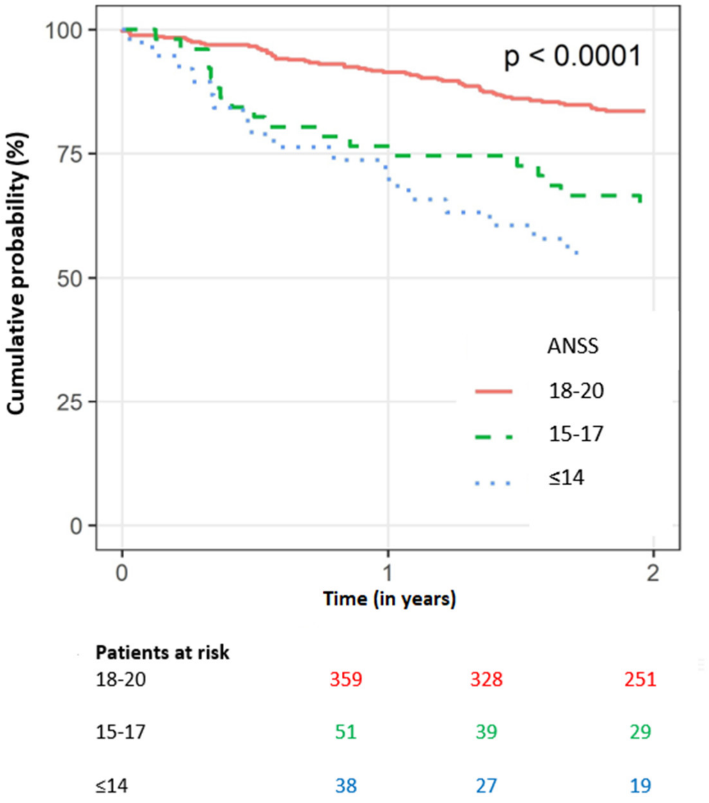

Supplementary Figure S1. Kaplan- Meier Survival curve for all-cause mortality for patients with LVEF ≤ 35% over time by admission Norton scale score. Numbers reflect patients at risk. p value <0.0001. ANSS= Admission Norton scale score

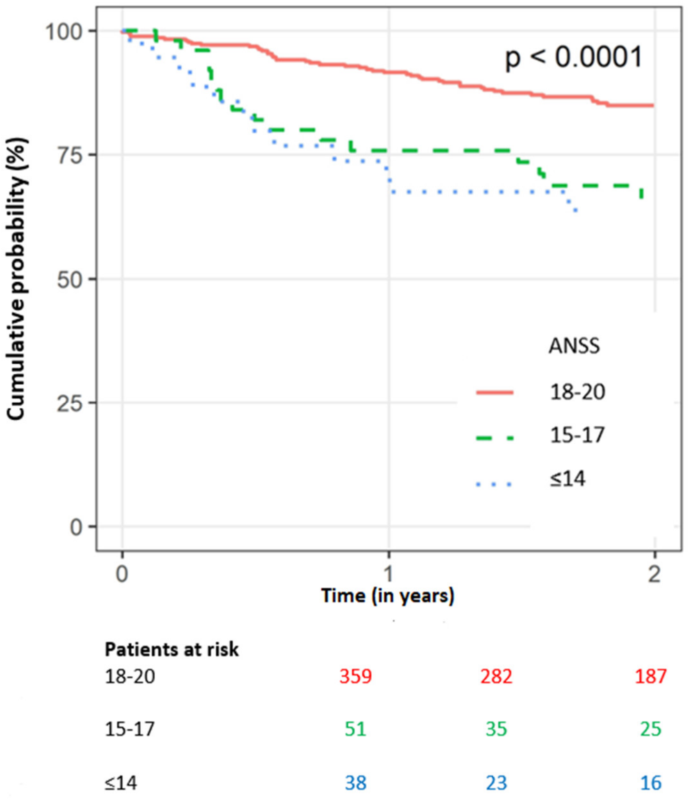

Supplementary Figure S2. Kaplan- Meier Survival curve for death without prior defibrillator therapy in patients with LVEF ≤ 35% over time by admission Norton scale score. Numbers reflect patients at risk. p value <0.0001. ANSS= Admission Norton scale score

Supplementary Figure S3. Kaplan- Meier Survival curve for all-cause mortality over time in high vs. low clinical risk patients with LVEF ≤ 35%, by admission Norton scale score. Numbers reflect patients at risk. ANSS= Admission Norton scale score. (**a**) High clinical risk patients: ≥ 70 years old and/or baseline serum creatinine values ≥ 1.5 mg/dL. p value = 0.00258; (**b**) Low clinical risk patients: <70 years old and baseline serum creatinine values <1.5 mg/dL. p value =0.163.

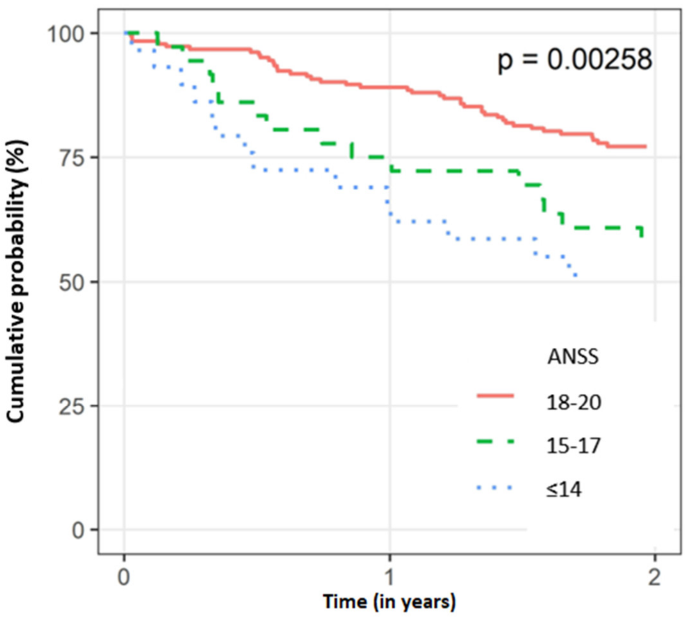

Patients at risk			
18-20	183	163	121
15-17	36	27	18
≤14	29	19	13

(**a**)

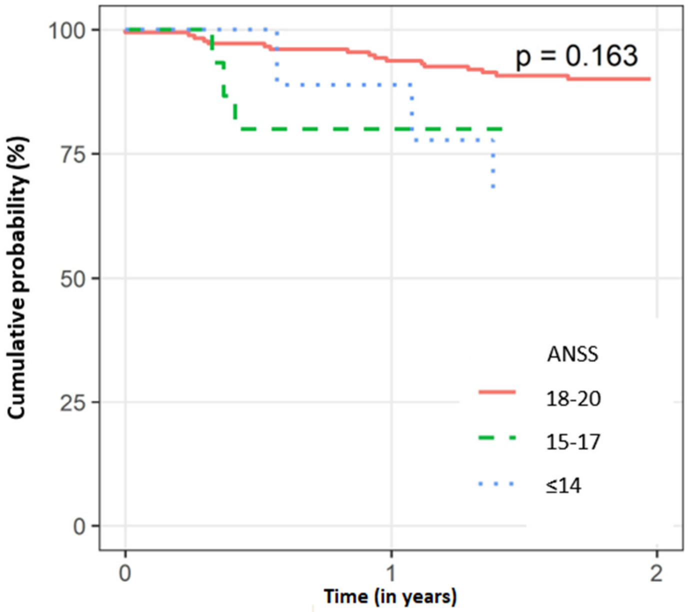

Patients at risk			
18-20	176	165	124
15-17	15	12	11
≤14	9	8	6

(**b**)

Supplementary Tables

Table S1. The Norton Pressure Sore Risk-Assessment Scale Scoring System

Item	Condition	Value
**Physical condition**	Good	4
	Fair	3
	Poor	2
	Very bad	1
**Mental condition**	Alert	4
	Apathetic	3
	Confused	2
	Stuporous	1
**Activity**	Ambulant	4
	Walks with help	3
	Chair bound	2
	Bedridden	1
**Mobility**	Full	4
	Slightly impaired	3
	Very limited	2
	Immobile	1
**Incontinence**	None	4
	Occasional	3
	Usually urinary	2
	Urinary and fecal	1

Table S2. Routine programming of implantable defibrillators in the Sheba medical center, Tel-Hashomer

Device	Programming
**Biotronik**	VT-zone (188 BPM), detection 36 cycled, redetection 30 cycles, Enhancements onset & stability. ATP X2 Bursts (8 x S1, Decrement: 10 ms, R-S1: 85%, Minimum Interval: 200ms) > Shocks: 8 x 40J<br>VF zone (240 bpm), Detection counter: 25 out of 31 cycles, ATP One Shot: Burst, shocks: 8 x 40J Redetection: 10 out of 14 cycles.
**Boston scientific**	VT zone (170 BPM), Monitor only<br>VF Zone (200 BPM) Duration 2.5 sec, Redetection 1 sec, Shock + Quick convert ATP
**SJM-Abbot**	VT Zone (185 BPM) No detection Enhancement. Duration – 6 sec Shock if unstable – On. ATP X2 Initial – 8, Coupling Interval – 88% without decrement, Maximum Shock Energy.
**Medtronic**	VT Zone (167 BPM), monitor only (VTNID 32/40), VF Zone (194 BPM) (VFNID 30/40, VFRNID 9/12) allow discrimination of SVT, Shocks 35J X6 +/- FVT Zone (250 BPM) ATPX8: Burst, R-S1 88%, Shocks 35J X6.

Table S3. Baseline characteristics of patients with LVEF ≤ 35% by admission Norton scale score.

Clinical characteristics	overall	ANSS ≤ 14	ANSS 15-17	ANSS 18-20	p value
**Study population**	n= 448	n= 38	n= 51	n=359	
**Age at procedure (years) ±SD**	67.8 ±11.6	72.6 ±12.9	70±12.4	67 ±11.2	0.005
**Female (%)**	62 (14)	4 (11)	9 (18)	49 (14)	0.612
**Prior myocardial infarction (%)**	198 (44)	15 (40)	23 (45)	160 (45)	0.815
**Congestive Heart failure (%)**	282 (63)	28 (73)	39 (77)	215 (60)	0.030
**Atrial fibrillation (%)**	136 (31)	12 (32)	16 (31)	108 (30)	0.976
**Prior CVA (%)**	51 (11)	10 (26)	11 (22)	30 (8)	<0.001
**prior TIA (%)**	14 (3)	0 (0)	1 (2)	13 (4)	0.415
**Dyslipidemia (%)**	224 (50)	19 (50)	23 (45)	182 (51)	0.734
**Currently on Dialysis (%)**	2 (0.4)	1 (3)	1 (2.0)	0 (0)	0.016
**Hypertension (%)**	246 (55)	22 (58)	28 (55)	196 (55)	0.939

**Diabetes mellitus (%)**	167 (37)	21 (55)	25 (49)	121 (34)	0.007
**Smoker (%)**	115 (26)	8 (21)	14 (28)	93 (26)	0.767
**BMI (kg/m²), ±SD**	27 ±5	26 ±4	27 ±5	27 ±5	0.125
**GFR MDRD (mL/min/1.73 m²), ±SD**	62 (31)	63 ±69	56 ±24	62 ±25	0.431
**Serum creatinine (mg/dL), ±SD**	1.3 ±0.6	1.4 ±0.6	1.4 ±0.7	1.3 ±0.5	0.220
**Hemoglobin (g/dL), ±SD**	12 (2)	11 ±2	12 ±2	12 ±2	<0.001
**Serum albumin (g/dL), ±SD**	4 ±0.5	3 ±0.6	4 ±0.5	4 ±0.4	<0.001
**LV Ejection fraction (%), ±SD**	25 ±7	27± 7	28 ±7	26 ±10	0.783
**ACE inhibitors (%)**	322 (72)	26 (68)	33 (65)	263 (74)	0.394
**Aldosterone antagonists (%)**	341 (76)	28 (74)	47 (92)	266 (74)	0.017
**Beta blockers (%)**	105 (55)	9 (24)	10 (20)	86 (24)	0.790
**Antiarrhythmics:**					

**Class IB (%)**	22 (5)	2 (5)	3 (6)	17 (5)	0.934
**Class IC (%)**	9 (2)	0 (0)	1 (2)	8 (2)	0.648
**Class III (%)**	232 (52)	22 (58)	24 (47)	186 (52)	0.599
**Salicylic acid (%)**	10 (2)	0 (0)	1 (2)	9 (3)	0.604

[2] Values are mean ± standard deviation, median [IQR] or n (%).
[3] ACE= angiotensin-converting enzyme; ANSS= Admission Norton scale score; BMI= body mass index; CVA= Cerebrovascular accident; GFR= glomerular filtration rate; ICD= implantable cardioverter defibrillator; LVEF= left ventricular ejection fraction; MDRD= Modification of Diet in Renal Disease; TIA= transient ischemic attack.
